# Cancer-Associated Fibroblasts Influence the Biological Properties of Malignant Tumours via Paracrine Secretion and Exosome Production

**DOI:** 10.3390/ijms23020964

**Published:** 2022-01-16

**Authors:** Martin Vokurka, Lukáš Lacina, Jan Brábek, Michal Kolář, Yi Zhen Ng, Karel Smetana

**Affiliations:** 1 Institute of Pathological Physiology, First Faculty of Medicine, Charles University, 120 00 Prague 2, Czech Republic; martin.vokurka@lf1.cuni.cz; 2 Institute of Anatomy, First Faculty of Medicine, Charles University, 120 00 Prague 2, Czech Republic; 3 BIOCEV, First Faculty of Medicine, Charles University, 252 50 Vestec, Czech Republic; 4 Department of Dermatovenereology, First Faculty of Medicine, Charles University and General University Hospital, 120 00 Prague, Czech Republic; 5 Department of Cell Biology, Faculty of Science, Charles University, 120 00 Prague 2, Czech Republic; jan.brabek@natur.cuni.cz; 6 BIOCEV, Faculty of Science, Charles University, 252 50 Vestec, Czech Republic; 7 Institute of Molecular Genetics, Czech Academy of Sciences, 142 20 Prague 4, Czech Republic; michal.kolar@img.cas.cz; 8 A*STAR Skin Research Labs (A*SRL)—Biopolis, Skin Research Institute of Singapore, 8A Biomedical Grove #06-06 Immunos Singapore, Singapore 138665, Singapore; yizhen_ng@asrl.a-star.edu.sg

**Keywords:** cancer microenvironment, cancer ecosystem, cancer-associated fibroblast, IL-6, exosome

## Abstract

Cancer-associated fibroblasts (CAFs) are an essential component of the tumour microenvironment. They represent a heterogeneous group of cells that are under the control of cancer cells and can reversely influence the cancer cell population. They affect the cancer cell differentiation status, and the migration and formation of metastases. This is achieved through the production of the extracellular matrix and numerous bioactive factors. IL-6 seems to play the central role in the communication of noncancerous and cancer cells in the tumour. This review outlines the role of exosomes in cancer cells and cancer-associated fibroblasts. Available data on the exosomal cargo, which can significantly intensify interactions in the tumour, are summarised. The role of exosomes as mediators of the dialogue between cancer cells and cancer-associated fibroblasts is discussed together with their therapeutic relevance. The functional unity of the paracrine- and exosome-mediated communication of cancer cells with the tumour microenvironment represented by CAFs is worthy of attention.

## 1. Introduction

Cancer ranks as a leading cause of death in many developed countries worldwide [1]. The growing burden of cancer incidence and mortality notably reflects the ageing of the population and changes in cancer risk factors. In positive or negative ways, many of these factors are associated with socioeconomic development. Importantly, cancer ranks as a leading barrier to increasing life expectancy in every country, according to the WHO [2].

Malignant tumours are highly complex tissues. In various ways, tumour morphogenesis is driven by mechanisms that are similar to those regulating the development of organs and tissues under normal physiological conditions [3]. To acquire a normal 3D structure, organs and tissues require a precise system of regulatory mechanisms. The normal architecture consequently contributes greatly to their correct function and maintenance in the long term. Therefore, structural and functional integrity is a critical prerequisite for the health of human body over its lifespan.

The process of malignant transformation is associated with an error in genetic information. This error may result from heredity or may be acquired during postnatal life. These acquired mutations may occur stochastically, by exposure to chemical/physical mutagens, due to viral infection, etc. Successful cancerogenesis usually requires many consecutively acquired mutations. Their conservation in the human genome may be facilitated by the failure of DNA repair mechanisms. At present, there are no doubts regarding the primary and critical role of these genetic factors in cancerogenesis. Cancer may arise from a single cell being transformed in a multi-step process.

Nevertheless, the tumour is not formed exclusively by these mutated cells. Moreover, the popular view of completely deregulated cancer growth seems to be somewhat naïve. Besides genetic defects, the malignant cell must be observed in and characterised by the context of the tissue environment. 

There are numerous noncancerous cells located in the vicinity of mutated malignant cells, collectively forming the tumours. This non-malignant component in tumours is most frequently referred to as tumour stroma. Of note, the non-mutated genetic status of stromal cells does not necessarily imply their full functional normality. In recent years, it has become a widely accepted concept by many cancer biologists that good cells may become cancerous because of their exposure to a bad environment [4]. This notion was preceded by classical studies on the importance of senescence in the course of cancerogenesis [5,6]. Later, this concept of a dangerous neighbourhood was extended into clinical oncology with therapeutic implications for the cell interactome [7]. Conversely, the importance and power of a normal environment was depicted in seminal works on xenotransplanted teratomas [8], even in the 1970s. Surprisingly, the microenvironment was able to completely overdrive the malignant behaviour of teratoma cells, leading to their incorporation into normal structures of the embryo without signs of malignancy. 

Therefore, the tissue microenvironment requires closer attention. Several principal stromal cell populations form the cancer cell niche. Namely, cancer-associated fibroblasts (CAFs) and various types of immune cells (e.g., cancer-associated macrophages) significantly influence the biological properties of cancerous cells. This may result in the maintenance of the low differentiation status of cancer stem cells, the increased proliferative capacity of cancer cells, and consequently their increased migratory capacity. These advantages contribute to clinically significant phenomena, such as the formation of lymphatic/distant metastases [9]. Cancer cells, in collaboration with their noncancerous counterparts in tumours, even influence the metabolic status through the production of biologically active factors with impact on the liver, striated muscle, and white fat that initiate wasting, resulting in the death of cancer patients [10]. 

These stromal components of tumours are the result of complex interactions across the tumour niche. With a certain level of hyperbole, the tumour can be seen as a selfish organ [11]. While normal organs execute their physiological functions, thus supporting the survival of the whole organism, the immature and poorly developed tumour expands selfishly at the expense of the organism. However, even at a rather primitive stage of their development, tumours still require vital regulations. Without tight stromal regulatory mechanisms, rapidly proliferating clones may very easily find themselves in a disastrous situation. Uncontrolled attempts to proliferate in the absence of proper metabolic resources could end in cell death. In a broader view, this scenario may remind us of the ecological phenomena of population extinction. 

To conclude, the inclusion of population biology and community ecology can be inspiring for cancer biologists. Thinking of tumours as organs composed of a plethora of mutually interacting populations may allow us to better understand the processes governing cancer development and progress.

## 2. Fibroblasts Participate in Organ Morphogenesis

Fibroblasts participate in the 3D architecture formation of virtually all organs in the human body. Fibroblasts also significantly influence their correct functions. Speaking broadly, fibroblasts are the versatile spindle-shaped cells of mesenchymal origin that are primarily capable of both extracellular matrix (ECM) synthesis and ECM resorption via released proteolytic enzymes. Besides the production and maintenance of ECM and related molecules, fibroblasts are potent producers of various small signalling soluble molecules, e.g., cytokines and chemokines. However, fibroblast morphology in various tissues seems to be relatively uniform; there is an apparent positional memory of fibroblasts. This is governed by the genetic imprinting of the homeobox (HOX) family of transcription factors [12]. Similar fusiform morphology thus does not imply identical functional properties in fibroblasts of various origins. 

The importance of fibroblast functions in tissues can be clearly evidenced in embryonic development. Surprisingly, fibroblasts act here quite uniformly in different tissues during their development. Stereotypically, the organ primordium, the epithelial bud, is surrounded by condensed mesenchymal cells in early development. The epithelium is usually separated from the mesenchyme by a thin sheath of connective tissue—the so-called basement membrane. This membrane excludes the direct physical contact of some populations; however, this membrane is permeable for small soluble molecules. The extensive exchange of molecules between the epithelium and mesenchyme across the basement membrane is essential for the successful development of the organ primordium. For example, this stereotypical model may be applied to the development of skin appendages, hairs, mammary gland, and also teeth [13,14,15]. Moreover, the early development of visceral organs, such as the lung [16], liver [17], and exocrine pancreas [18,19], follows a very similar morphogenetic scenario. As summarised in these review articles, the exchange of molecules, such as members of the BMP and FGF growth factor families, between the epithelium and mesenchyme is indispensable. Further, other factors, such as members of the TGF-β, IGF, and Wnt family ligands, as well as EGF and HGF, must also be highlighted in this context. The interaction of these factors via the appropriate membrane-bound receptors initiates the expression of specific transcription factors that control the genetic programme of the particular cell type differentiation, finally leading to organ development.

Relevant to the skin structure, several populations of fibroblast lineages have been identified in the last few years; mouse models proved to be particularly useful for that [20,21]. Papillary and reticular fibroblasts show different morphologies and molecular markers in vivo and in vitro, and they can also differ functionally. A very prominent fibroblast population is also found in the dermal papilla of hair follicles [22]. There is increasing evidence that deeply located fibroblasts present at the border with adipose subcutis also represent a very different subset of dermal fibroblasts [23]. Single-cell sequencing methods proved to be very useful for unravelling cell heterogeneity. However, the single-cell analysis-based literature on human skin remains relatively scarce [24,25]. Moreover, these transcriptomics-identified markers may not be easy to link with the fibroblast subpopulation markers necessary in diagnostic pathology. Additionally, the extrapolation of mouse data to humans is not straightforward because murine and human skin differ significantly across many morphological and functional features.

## 3. Fibroblasts as a Fundamental Bioactive Part of the Majority of Tumour Types

Tumours represent complex multicellular tissue/organ-like structures. The tumour cell type composition is known from histological research. Moreover, intercellular interactions could be easily observed and described using the established terms and principles of ecology. This approach facilitates a better understanding of the relation between the cancerous and noncancerous cell populations in primary tumours.

Intercellular interactions of various populations in the tumour also influence the spreading of cancer cells and the formation of metastases [26]. Even a highly malignant cell requires a safe harbour [27]. While the process of tumour formation and progression is relatively slow, it is possible for cancer cells to gradually gain control and rearrange the components of the tumour microenvironment through mutual and dynamic communication [28]. However, the migrating or circulating cancer cells require vital support to colonise their new niche and form metastases. It is very likely that the rate of success of these individual malignant cells at forming a new metastasis is remarkably low [29]. It is a well-fitting cellular embodiment of the classical seed and soil theory coined by Stephen Paget [30]. However, the soil does not passively accept the seeds. The failure of individual cells here is not due to the lack of their malignant features, but it is likely attributed to their meeting a non-permissive environment populated by non-cooperative cell types. With clinical relevance, most of the deaths in oncology may be attributed to metastatic disease [31]. 

The successful development of cancer in a patient’s body is, thus, in terms of Darwinian biology, a result of the co-evolution of the cancerous and noncancerous cells of the tumour. It has been well documented in many types of cancer that multiple steps of carcinogenesis leading to the cumulative effect of consequently acquired mutations (e.g., loss of key tumour suppressor genes and the overexpression of important oncogenes) in an individual cell will cause a normal cell to become a cancerous cell [32]. This ongoing evolution influences the fibroblasts in their vicinity. In this light, fibroblasts that positively influence the growth of cancer cells and their migration are of primary importance [33]. Consequently, fibroblasts modify the local immune response and thus stimulate the cancer-supporting function of the immune cells infiltrating the tumour site [34,35,36].

There is limited empirical data available on this interaction during the tumour progression. The theoretical literature in evolutionary studies usually highlights that interaction-dependent population size changes are likely the key to the full understanding of these co-evolutionary dynamics [37]. Thus, a more realistic view of the complex nature of tumour–stromal interactions is urgently needed. 

It is likely assumption that the quantity of stromal cells is cancer type specific. It is well known that the proportion of CAFs and other stromal cells differs according to the type of solid tumour [38]. In some types of cancer, CAFs are numerous and extensively produce the extracellular matrix. This is a prominent feature in, e.g., highly malignant ductal adenocarcinoma of the pancreas, referred to as the desmoplastic type of tumour [39,40]. On the other hand, the proportion of stromal cells is usually somewhat modest in cutaneous malignant melanoma [34] or glioblastoma [41]. Despite their sparse quantity, the CAFs observed in these tumours are highly biologically active. Our knowledge remains sparse for other types of solid malignancies of non-epithelial origin, e.g., in sarcomas. Except for Ewing sarcoma [42] and osteosarcoma [43], the role of CAFs in sarcomas of different origins is unclear. Further, CAFs have not been extensively studied in haematological malignancies so far. However, there is evidence that cells with the properties of CAFs have been identified in both acute and chronic lymphocytic leukaemia, Hodgkin and follicular lymphoma, and multiple myeloma [44]. 

### 3.1. Origin of CAFs

The fibroblast populations in the vast majority of human body structures originate in the mesoderm germ layer. However, fibroblasts of the facial part of the head and part of the neck are raised from the neural crest; therefore, they are also referred to as ectomesenchyme [45,46]. Interestingly, there are significant differences in transcriptomic profiles between mesoderm-originated and ectomesenchyme-originated fibroblasts. This significant distinction is preserved even in adult life, as evidenced at the whole-genome scale [47]. 

As mentioned earlier, the cancer cell population undergoes the progressive multistage genetic alteration necessary to acquire fully malignant features. However, the CAFs are not primarily driven by acquired mutations. It is a vitally important task for proliferating malignant clones to initiate communication with the surrounding tissue in a timely manner. Without a permissive or even supportive environment, the clone will very likely not survive. Via this dialogue [48], cancer cells are able to recruit or even corrupt [49] the various cells around them, including tissue fibroblasts. In recent years, it has become widely recognised that epigenetic alterations are essential for regulating CAF bioactivity [50]. This local recruitment is the most widely recognised theory of the origin of CAFs, and it was also suggested as a potential target for therapeutic interventions in cancer [51].

However, CAFs may theoretically be descendants of cell types other than local fibroblasts. The epithelial–mesenchymal transition (EMT) represents a critically important developmental mechanism [52]. However, it is also one of the leading mechanisms in tumour spreading; it is necessary to initiate metastasis. Notably, epithelial cells undergoing EMT newly acquire the mesenchymal phenotype. This mesenchymal state is also associated with the capacity of cells to migrate. Therefore, it is a very attractive hypothesis that CAFs may originate from malignant cells via EMT [53,54]. This would represent a significant advantage for malignant cells, because the newly formed tumour would become self-sufficient at producing supportive stromal structures. This hypothesis is mainly supported by the observation of mutations in CAFs. However, should CAFs really originate from the tumour cell population, there would have to be a significant overlap in their mutational repertoire. As opposed by others, mutations in CAFs can also be explained by the broad-field cancerisation of mutagenic agents affecting both cancer cells and the surrounding mesenchyme [55,56]. In our own experience, we have not observed the concordant mutation of, e.g., BRAF V600E in melanomas and in isolated CAFs [57]. In other experiments, human cancer cells grafted to immunocompromised mice developed tumours, while the stroma was of host origin, as confirmed by a species-specific antibody recognising the human and not murine vimentin [58]. Based on these summarised data, the formation of CAFs from cancer cells via EMT in vivo is likely not a principal mechanism.

Other candidate cell types hypothesised as precursors of CAFs and factors stimulating their formation [59,60,61,62,63,64,65] are included in Table 1. Members of the TGF-β family seem to be the most frequently discussed in this context as inducers of the transition of precursor cells to myofibroblasts [66].

Significantly, a great deal of attention in the last few years has also been dedicated to mesenchymal stem cells (MSCs) and their ability to migrate to cancer sites. Besides the many unique properties of MSCs, these lowly differentiated cells can also differentiate to CAFs and gain their full properties. This behaviour, however, also substantiated the proposal of a new therapeutic strategy based on the loading of MSCs with oncolytic viruses to destroy cancer cells [67,68].

### 3.2. Markers of CAFs

**Figure 1:**
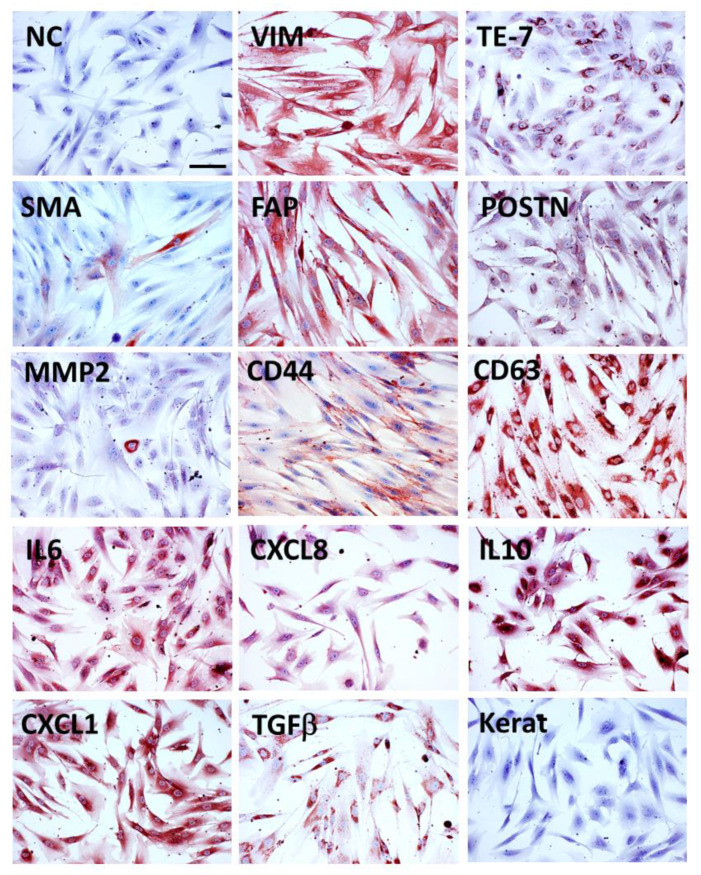
CAFs isolated from human cutaneous basal cell carcinoma (immunocytochemical analysis, red AEC product indicates positive reaction, NC—negative control with isotype antibody, bar represents 100 μm). CAFs are expressing intermediate filament protein vimentin (VIM), and specific mesenchymal marker TE-7. SMA was detected only in a minority of cells, while FAP was present universally, and periostin (POSTN) was also found in virtually all cells. MMP2 was positive only in sporadic cells. CD44 (hyaluronic acid receptor) was widely expressed with marked positivity on the periphery of cells. Tetraspanin CD63 is associated with the membranes of intracellular vesicles and was detected in all cells as a distinct granular signal. CAFs produce high quantities of interleukins and chemokines, such as IL-6, CXCL-8, IL-10, CXCL-1, and TGF-β. Despite using pan-keratin reactive antibodies, no keratin positivity was observed in CAFs.

Diagnostic pathology remains a vitally important discipline in any biomedical research, including on cancer. Immunohistochemical and molecular analysis further enhances our insight into the frequently subtle changes modifying the disease progression. Unfortunately, no specific marker reliably distinguishing CAFs from other fibroblasts has been discovered so far. The frequently mentioned notorious marker of tumour stroma expressed in CAFs is α-smooth muscle actin (SMA). However, not all of the fibroblasts in the tumour stroma in vivo or fibroblasts isolated from tumour stroma in vitro express this protein (Figure 1).

However, SMA expression is not a sufficient marker for mature myofibroblast identification [69]. The cytoskeletal distribution of SMA and the formation of stress fibres should also be considered. In fully mature myofibroblasts, the neo-expression of SMA-positive stress fibres is present. The absence of SMA within these fibres facilitates the distinction of proto-myofibroblasts from mature myofibroblasts. However, the significance of these steps of myofibroblast maturation and their role in tumour biology remains unclear. It has been suggested that the inhibition of SMA polymerisation could also have some therapeutic prospects [70].

**Figure 2:**
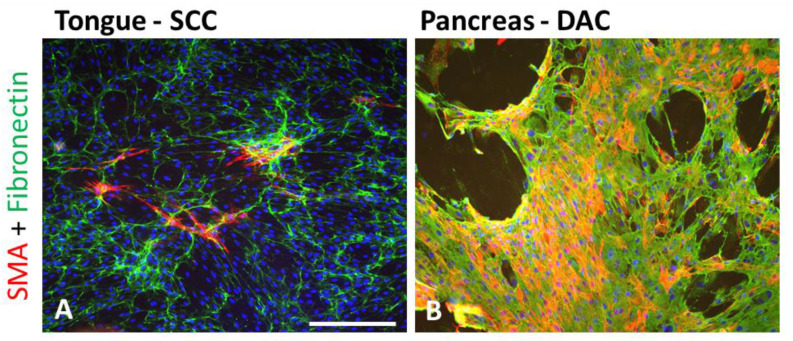
CAFs isolated from squamous cell carcinoma (SCC) of the tongue (**A**) and ductal adenocarcinoma (DAC) (**B**) of the pancreas (desmoplastic form) express SMA (red) and produce fibronectin (green). Bar represents 100 μm.

For the sake of practicality, SMA expression across different types of tumours seems to be quite universal. However, the extent of the myofibroblastic population observed in various tumours varies greatly (Figure 2).

Therefore, the identification of additional protein markers reflecting the activation of CAFs or their different origin may be helpful for recognising CAFs in diagnostic tissue sections or in cell culture [71,72,73,74]. These markers are listed in Table 2.

Some of these markers, such as FAP, can be potentially employed in theranostic applications as a diagnostic and therapeutic tool [75,76].

### 3.3. Heterogeneity of CAFs

Recent progress in molecular biology methods, such as single-cell RNA sequencing, have facilitated deeper insights into tissue population heterogeneity. Using this powerful tool for distinct tumours and experimental situations, the subpopulations in cancer stroma, including CAFs, can be analysed in detail. Dermal fibroblasts cultured in 3D heterogeneous spheroids with melanoma cells demonstrated their ability to gain CAF-like properties. Notably, sun-damaged fibroblasts from aged donors transformed more readily than juvenile control fibroblasts [25]. Even in this simplistic 3D model, fibroblasts represent a heterogeneous population. One part of the CAFs predominantly produced the extracellular matrix (ECM). This is primarily consistent with the expected fibroblast role in the dermis. In contrast, another subpopulation predominantly produced inflammatory mediators. These factors, namely IL-6 and CXCL-8, are, to a great extent, produced in fibroblasts, with only a negligible quantity of ECM. Another subpopulation of fibroblasts observed in this model was driven by the influence of TGF-β signalling [25]. Similarly, other authors have demonstrated the heterogeneity of CAFs in clinical samples from different tumours or in animal experiments [75].

It was suggested that CAFs could be further subclassified into (a) immune CAFs that produce inflammatory mediators, (b) desmoplastic CAFs that secrete ECM, (c) contractile CAFs that have a high expression of SMA, and finally (d) aggressive CAFs that occur in aggressive tumours [76,77,78]. Notably, TGF-β signalling can drive several of these phenotypes, e.g., contractile CAFs and desmoplastic CAFs. Interestingly, the comparison of CAF heterogeneity demonstrated some similarities across different types of tumour [79,80,81]. This confirms earlier observations of the functional similarities among CAFs of various origins in vitro [82]. Further, it suggests that the transcriptional profile of CAFs can influence the biological properties of cancer in general, and it is worthy of attention and potentially also targeting. Based on these observations, it seems likely that a detailed analysis of CAF subtypes can be employed to refine diagnostics in the future. The practicality of this concept was tested in patients suffering from aggressive adenocarcinoma of the pancreas [83].

### 3.4. Role of CAFs in Cancer Biology

The cancer microenvironment, including CAFs, is to some extent similar to the site of a healing wound. SMA-expressing myofibroblasts are responsible for wound contraction and the production of bioactive factors that facilitate tissue integrity recovery [9,84,85]. The role of CAFs is quite similar to the role of myofibroblasts in the wound, but their functional activity is not time restricted [86]. It is known that wound healing and cancer share many important signalling pathways [87] and differ in temporal regulation. Sadly, we lack the proper understanding of the mechanism representing the master switch in CAFs.

It is known that CAFs can influence the differentiation status of cancer cells and the normal epithelium. Using CAFs in various in vitro models, it was demonstrated earlier that normal epithelial cells express markers characteristic of the immature cells of low differentiation status [88,89]. Moreover, when CAFs were co-cultured with other fibroblasts, these fibroblasts underwent phenotypic changes, and the differentiation potential of exposed fibroblasts was consequently comparable to mesenchymal stem cells [86].

Low differentiation status maintenance may be a critical advantage for tumour cells during, e.g., chemotherapy. The stem cell populations, including cancer stem cells, use several molecular transport mechanisms, causing their resistance to xenobiotics, including chemotherapy. Therefore, the maintenance of stemness may be a major cause of treatment failure. Besides that, the tumour microenvironment forms a safe harbour for cancer cells. The desmoplastic tumour niche may physically represent a site where cancer cells are exposed only to the tolerable doses of anticancer drugs [90].

Mediators of the intercellular communication between cancer cells and noncancerous cells, including CAFs, can also leak from the primary tumour site. Thus, CAFs can participate in the formation of premetastatic niches in distant body parts [10,57,91]. CAFs also significantly influence the migration of cancer cells, such as melanoma and glioblastoma cells, in vitro [92,93], resulting in a consequently increasing probability of colonising the new permissive site.

With an emphasis on the systemic clinical effect, elevated levels of these mediators are responsible for cancer patient wasting and preterminal cachexia [10,92,93]. As the activity of CAFs seems to be quite conserved, despite their origin and type of cancer, this seems to be worthy of targeting and therapeutic intervention.

#### 3.4.1. Production and Remodelling of ECM

In the most classical view, fibroblasts are responsible for the maintenance of the structural integrity of connective tissues. Similar to other types of fibroblasts, CAFs also produce molecules of ECM, such as collagen, hyaluronic acid, fibronectin, periostin, and tenascin [94,95,96,97,98,99,100,101]. ECM production by CAFs seems to be more extensive than in normal fibroblasts, and it even reflects the severity of the disease.

On the other hand, stromal cells, including CAFs, also produce proteases [102,103]. This complexity of dynamic ECM production and degradation seems to be essential for tissue healing, but it is crucial for tumour spreading and metastasis formation as well [104,105,106]. In tissue samples, CAFs are usually observed at the leading edge of collectively migrating cancer cells, where they prepare a corridor, facilitating cancer cell migration and consequent metastatic spread [107].

#### 3.4.2. Production of Bioactive Factors with Emphasis on IL-6

CAFs produce numerous bioactive factors, which have been reviewed extensively in numerous articles [10,108,109,110,111,112]. When comparing different tumour types, IL-6 seems to be widely expressed by CAFs in many, if not in virtually all, cancer types [51,113]. As shown in these articles, IL-6 participates in the control of the low level of differentiation of cancer cells and the regulation of their migratory activity. The systemic effect of IL-6 leads to the wasting of cancer patients. Further, after passing through the blood–brain barrier, IL-6 may have behavioural effects and induce depression [114].

As was demonstrated in vitro, the production of IL-6 by CAFs is precisely controlled in the co-culture of cell lines from the ductal cancer of the pancreas and CAFs isolated from the same cancer type [39]. However, IL-6 should also be seen as a principal component of the senescence-associated secretory phenotype in many cells [115]. Indeed, IL-6 production is tightly associated with ageing, and normal dermal fibroblasts from aged persons are thus functionally quite close to CAFs [116]. A very similar observation was reported in the heterogeneous spheroids prepared from melanoma cells and juvenile/adult dermal fibroblasts [25].

To elicit a cellular response, IL-6 must bind to and be recognised by a receptor [117]. Mechanistically, IL-6 is bound to IL-6R and associated with a signal transducer called glycoprotein GP-130. IL-6R is docked to the cell membrane; however, it can be proteolytically cleaved from the membrane and, thus, it becomes soluble (sIL-6R) and can circulate in body fluids. Both types of IL-6 receptors require cooperation with glycoprotein gp130 to initiate signalling via the phosphorylation of other interacting molecules. The effects of the membrane-bound and soluble IL-6R forms are not identical [118,119,120], and it is necessary to distinguish classical versus trans-signalling effects. Therefore, the action of IL-6 is understood to be context-dependent and highly complex.

The inhibition of IL-6 signalling via receptor interference can be achieved by, e.g., monoclonal antibodies. In clinics, tocilizumab was proven to be a highly effective therapy in rheumatoid arthritis; however, it failed to be sufficient as a cancer therapy [119]. Nevertheless, current cancer therapy is frequently based on combinations, and the simultaneous targeting of several mechanisms can be beneficial and affordable. Therefore, the targeting of IL-6 along with the targeting of other molecules seems to be worthy of attention [111,113,120]. We can hypothesise as to which factors should be hit along with IL-6. It was suggested elsewhere that the targeting of IL-6 and CXCL-8 could be promising [121]. More recently, Ham and co-workers [122] confirmed the prominent role of IL-6 and TGF-β in tumours of the gastrointestinal tract, where these two factors induce resistance to chemotherapy and actinotherapy.

## 4. Tumour Cell-Derived Exosomes in Cancer Biology

### 4.1. Definition of Exosome

**Figure 3:**
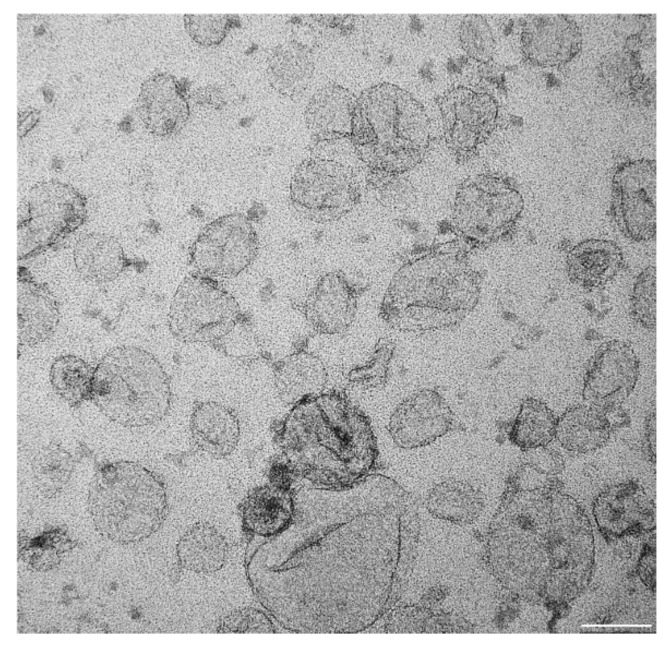
Air-dried exosomes prepared from G361 melanoma cells cultured in exosome-depleted calf serum. Exosomes were adsorbed on a formvar membrane, fixed with 1% glutaraldehyde and contrasted by uranyl acetate, and detected using an electron microscope. The bar is 50 nm.

Exosomes are small vesicles of 30–100 nm in diameter formed by a double lipid bilayer with the presence of several proteins, including tetraspanins—namely, CD9, CD63, and CD81 (Figure 3). Exosomes are released from cells via multivesicular body production. Their cargo can potentially be presented by almost 41,000 proteins, 5000 mRNA, and 3000 microRNAs (miRNAs). These data are continually updated in the exosome protein, RNA and lipid database (http://exocarta.org/; accessed 20 December 2021). Exosomes thus represent a powerful tool for the vertical transfer of information in a highly concentrated form [123,124,125].

### 4.2. Effect of Cancer Cell-Derived Exosomes (CaExos) on the Function of CAFs

**Figure 4:**
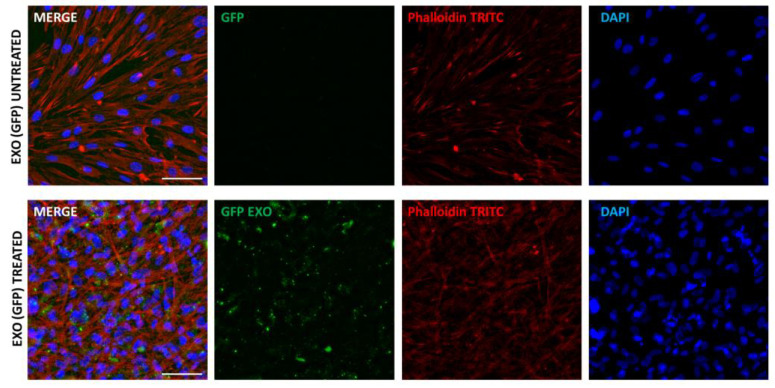
CAFs from cutaneous basal cell carcinoma were treated by commercially available GFP-tagged exosomes for 12 h. Cells were consequently fixed by paraformaldehyde, permeabilised, and counterstained using phalloidin-TRITC (to show actin in the cytoplasm) and DAPI (to show DNA in the nuclei). Imaging was performed with a Leica Thunder Imager using the computational clearing technique to efficiently differentiate between the signal and background to visualise the distribution of GFP-tagged exosomes in CAFs. Bar represents 100 μm.

CaExos participate in the formation of the tumour microenvironment. In the broader sense, CaExos can facilitate the formation of the premetastatic niche in different types of tumours, including melanoma [126,127,128]. Exosomes can modulate various cells to prepare the microenvironment for cancer cell docking [129]. The majority of studies addressed questions regarding the role of exosomes in the mutual communication between cancer cells. However, the effect of CaExos on CAFs has not been so extensively elucidated. Indeed, CAFs can bind and internalise exosomes (Figure 4) and be subject to regulation by cancer cells.

**Figure 5:**
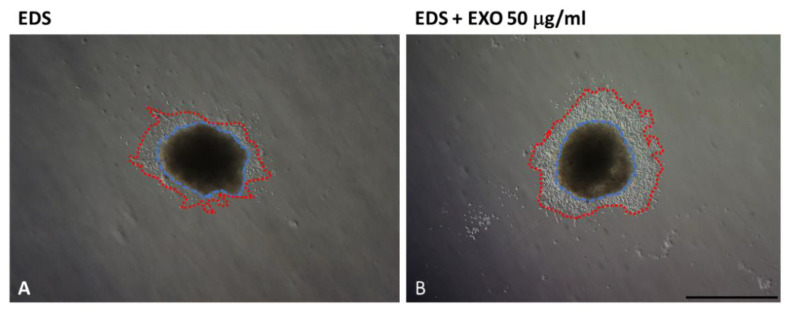
Melanoma cells migrate from the heterogeneous spheroids prepared from melanoma CAFs and melanoma cells (**A**,**B**). Melanoma-derived CaExo treatment facilitated migration (**B**). The blue circle demonstrates the position of the original spheroid, and the red circle highlights the leading edge of migration. To follow only the influence of melanoma-derived exosomes, the 3D collagen gel and culture medium contain only exosome-depleted serum. Bar represents 1 mm.

Melanoma-derived CaExos negatively influence the adhesion and proliferation of both the normal dermal fibroblasts and melanoma CAFs in a dose-dependent manner [130]. This finding reflects well the secretory senescent phenotype of the CAFs observed earlier [131,132]. This cellular phenotype is associated with the upregulation of miR-335 [133]. On the other hand, the in vitro invasiveness of both tested types of fibroblasts, i.e., normal dermal and CAFs, is stimulated in 3D collagen gels by the melanoma-derived CaExos. This may be understood to be a model of the invasive front mentioned earlier. Further, CaExos (melanoma) also stimulated the migration of melanoma cells from heterogeneous spheroids prepared from the melanoma cells and melanoma-isolated CAFs (Figure 5) in collagen gels [130].

The expression profiles of normal dermal fibroblasts and melanoma-derived CAFs significantly differ after exposure to melanoma-derived CaExos. CAFs increase the expression of inflammation-promoting factors, including IL-1, IL-6, and CXCL-8, after CaExo treatment at the mRNA level, and a similar trend was also confirmed at the protein level. The normal dermal fibroblasts are also activated by CaExos, but their expression profile differs. In detail, normal dermal fibroblasts exposed to CaExo treatment mainly increased the expression of thrombospondin-1 at the protein level—this factor is important for tissue healing. Importantly, changes in the expression of IL-6 and CXCL-8 were insignificant at the protein level, although there were changes at the mRNA level [130]. The participation of melanoma-derived CaExos in the inflammation-supporting microenvironment was also observed by Lahav and co-workers [134]. The effects of CaExos on normal fibroblasts or mesenchymal stem cells as their precursors were also described in other model situations where normal cells can consequently acquire the properties of CAFs [135,136,137,138,139,140,141,142,143]. CaExos are also able to stimulate the endothelial cell–mesenchymal transition and the formation of CAFs from these precursors [144]. This activity seems to depend on the exosome cargo, mainly on miRNAs (Table 3).

### 4.3. CAFs Produce Exosomes That Influence the Function of Cancer Cells

Cancer cells produce exosomes that significantly activate CAFs. A certain reciprocity can be easily expected here, and activated CAFs produce exosomes with a very significant impact on cancer cells. The effect of exosomes is enhanced by the low pH and hypoxia—these features are typical of the majority of solid tumours, independently of their origin [145,146,147]. The exosomes produced by CAFs notably influence the properties of cancer cells via a broad panel of mechanisms. This fosters stem cell-like properties in cancer cells, maintains their low differentiation status, and regulates their proliferation and migration. This is a base for the formation of metastases and resistance to chemo/actinotherapy. CAF-derived exosome efficiency mainly depends on the specific miRNAs in their cargo [73,122,136,148,149,150,151,152,153,154,155,156,157,158,159,160] (summarised in Table 4).

### 4.4. Systemic Effects of Exosomes—Role in Cancer Wasting and Cachexia

Cancer wasting and cachexia are present in many patients in the terminal phase of malignant disease [161]. The terminal progression of the disease is associated with the production of cytokines, such as IL-6 and TNF-α. These mediators are produced by the cells of the cancer ecosystem [113] and leak to the circulating body fluids. Cancer cachexia is therefore characterised by systemic inflammation. Exosomes can also significantly participate in this deadly complication [162,163], mainly through their effect on the production of IL-6 [130]. However, non-coding RNAs seem to be critically important in the regulation of the metabolic changes leading to cachexia [163]. Mechanistically, skeletal muscle wasting as part of tumour-related cachexia in an animal model is associated with the presence of miR-125b-1-3P in the exosomes [164]. Similarly, structural and functional changes of white adipose tissue were observed in cachectic patients. This browning of adipose tissue may be linked to the effect of miR-146b-5p carried in exosomes [165]. This insidious syndrome dramatically impacts the quality of the remainder of the patient’s life, but is also associated with poor responses to therapy and decreased survival [166].

## 5. Conclusions

Tumours involve a complex cellular ecosystem with a multitude of intercellular interactions ongoing simultaneously. Malignant cells represent the principal population of tumours; however, stromal components forming the microenvironment cannot be neglected. Among many others, CAFs are one of the most common noncancerous cell types involved in forming tumours. CAFs are not homogeneous, and several functional subtypes can be distinguished in this cell population of mesenchymal origin. Their function is influenced by the cancer cells; however, CAFs also reversely influence the malignant population in many ways. CAFs continuously shape the landscape of the tumour microenvironment. Structurally, CAFs, as with any typical fibroblasts, produce and remodel ECM. However, their important function also lies in the paracrine production of a wide scale of bioactive factors. Significantly, CAFs produce molecules supporting the local inflammatory milieu that seems to be indispensable for cancer cell proliferation and invasiveness.

Moreover, the inflammatory factors can leak from the primary tumour site via vessels and can also have a systemic effect. This can contribute to the formation of a premetastatic niche in distant tissues, which is critically important for successful metastatic spread. Concerning the whole body of the cancer patient, these factors also actively participate in the induction of cancer wasting and cachexia, which affects the patient’s treatment and decreases survival rates.

**Figure 6:**
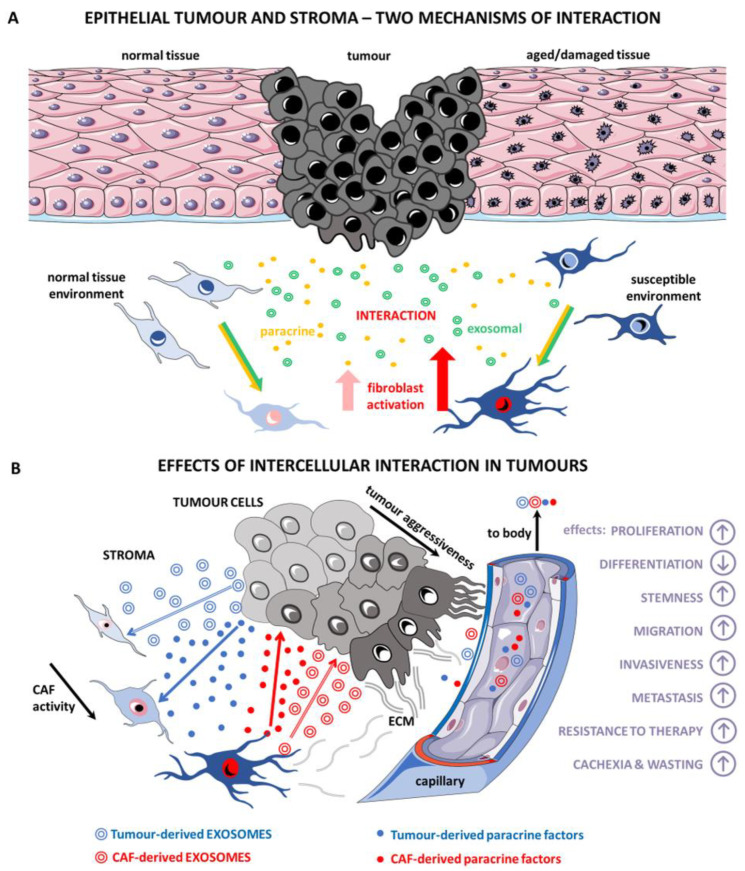
Cancer cells interact with stromal CAFs via the paracrine secretion of bioactive substances, such as cytokines/growth factors, and also release exosomes. This leads to the formation of the permissive microenvironment necessary for tumour progression. The response of normal fibroblasts and aged/damaged fibroblasts to this stimulation is similar, but not identical (**A**) Tumour cells and CAFs use both modes of interaction (exosomal and paracrine) mutually in communication across the landscape of the tumour. These stimuli influence the biological properties of cancer cells, including the formation of metastases, and even exhibit systemic effects, such as wasting and cachexia (**B**) Graphics based on free Servier Medical Art image bank available at smart.servier.com. Servier Medical Art is licensed under a Creative Commons Attribution 3.0 Unported License.

Exosomes represent a versatile tool for intercellular communication. CaExos facilitate the transition of precursor cells to fully cancer-promoting CAFs. CaExos significantly activate CAFs via the transmitted cargo or via miRNAs and proteins, such as TGF-β. Further, CaExos stimulate CAFs to produce a broad array of pro-inflammatory factors, e.g., IL-6 and CXCL-8. Conversely, exosomes produced by CAFs also have a distinct effect on cancer cells, which is mediated by the action of a wide panel of miRNAs in the exosomal cargo. Collectively, these miRNAs directly affect the important functions of cancer cells, such as differentiation, proliferation, and migration (Figure 6). These miRNAs also consequently induce resistance to chemotherapy and actinotherapy. This mechanism contributes to the failure of oncological therapy. Moreover, these miRNAs directly impact the metabolism of muscle fibres and adipocytes and, thus, adversely modify the terminal course of malignant disease: cancer wasting and cachexia.

This review demonstrates the functional unity of a paracrine and exosome-mediated dialogue between the cancer cells and the microenvironment represented by CAFs in the context of a tumour. In many clinical trials, the therapeutic intervention is based on monotherapy or targeting a single bioactive factor. Relevant to, e.g., IL-6 or its receptor, this therapeutic targeting was not as successful as expected. Perhaps targeting both subsystems of intercellular communication inside the cancer ecosystem, paracrine and exosome-mediated communications, simultaneously could be more fruitful.

## Figures and Tables

**Table 1:** Cell types as potential precursors for CAF formation.

Cell Type	Factors Inducing CAF Formation from Precursors	Note
Resident fibroblasts	IL-1β, TGF-β, FGF, PDGF	
Stellate cells	SDF1, TGF-β, PDGF	
Mesenchymal stem cells	SDF1, TGF-β, HDGF, FGF	
Adipocytes	TGF-β	
Mesothelial cells	TGF-β	
Fibrocytes	SDF1, TGF-β, FGF, IL-12, IFNγ	Novel fibrocyte subset circulating in peripheral blood
Pericytes	SDF1, TGF-β, PDGF	
Epithelial cells	TGF-β	Epithelial–mesenchymal transition is necessary
Endothelial cells	IL-1β, TGF-β, PDGF, TNFα	Endothelial–mesenchymal transition is necessary

**Table 2:** Markers of CAFs according to their origin.

Origin	Marker
Resident local fibroblasts	**SMA**, type I collagen, CCL2, RAB3B, tenascin C, periostin, podoplanin, S100A4, CD74
Mesenchymal stem cells	DDR2, FSP-1, CXCL12, vimentin, **SMA**, calponin-1, PDGFRα, periostin, CD90, podoplanin
Adipocytes	FSP-1, **SMA**, FAP, ASC-1
Endothelial cells	CD31, FSP-1, **SMA**, TGF-β2
Pericytes	PDGF-BB, NG2, FSP-1, **SMA**

Legend: SMA is the most common marker and therefore is in bold.

**Table 3:** Example of CaExo cargo molecules activating fibroblasts/mesenchymal stem cells/CAFs.

Type of Cancer	Molecule
Melanoma	miR-21, miR-155-5p, miR-210, miR-211, miR-222, long noncoding RNA Gm26809
Chronic lymphocytic leukaemia	miR-146a
Breast cancer	miR-146a
Head and neck squamous cell carcinoma	TGF-β1

**Table 4:** Effect of CAF-derived exosomes on cancer (selected examples).

Type of Cancer	Molecule	Effect
Breast	miR-92 miR-500a-5p miR-580-5p	Deregulation of PD-L1 Cancer cell proliferation and migration Cancer cell stemness
Gastrointestinal tract	miR-24-3p, miR-92a-3p, miR-135b-5p, miR-522, miR-590-3p	Cancer cell proliferation, angiogenesis, resistance to chemotherapy
Liver	miR-150-3p	Hepatoma cell migration
Lung cancer	miR-130a, miR-142-5p, miR-369	Deregulation of PD-L1, cell migration, resistance to cisplatin
Malignant lymphoma	miR-4717-5p	Resistance to anti-pyrimidine drugs
Urinary bladder cancer	Long intergenic non-protein coding LINC00355	Resistance to cisplatin
Prostate	miR-423-5p	Resistance to chemotherapy
Melanoma	miR-21, miR-155-5p	TGF-β production, tumour progression, angiogenesis support

## Data Availability

Data are available in authors’ archive.

## Abbreviations

ASC-1	Neutral amino acid transporter SLC7A10
CAFs	Cancer-associated fibroblasts
CaExos	Cancer cell-derived exosomes
CCL	Chemokine (C-C motif) ligand
CXCL	Chemokine (C-X-C motif) ligand
DDR2	Discoidin domain receptor 2
ECM	Extracellular matrix
EGF	Epidermal growth factor
EMT	Epithelial–mesenchymal transition
FAP	Fibroblast activation protein
FGF	Fibroblast growth factor
FSP1	Fibroblast-specific protein 1
HDGF	Hepatoma-derived growth factor
HGF	Hepatic growth factor
IGF	Insulin-like growth factor
IL	Interleukin
KERAT	Keratin
miR	microRNA
MMP	Matrix metalloproteinase
NG2	Neural/glial antigen 2
PDGF-BB	Platelet-derived growth factor BB
PDGFRα	Platelet-derived growth factor receptor α
PD-L1	Programmed cell death ligand 1
RAB3B	Ras-related protein Rab-3B
SMA	α-Smooth muscle actin
TGF	Transforming growth factor
TNFα	Tumour necrosis factor α
WNT	Wingless and Int signalization
